# *Lactobacillus Acidophilus/Bifidobacterium Infantis* Probiotics Are Beneficial to Extremely Low Gestational Age Infants Fed Human Milk

**DOI:** 10.3390/nu12030850

**Published:** 2020-03-22

**Authors:** Ingmar Fortmann, Janina Marißen, Bastian Siller, Juliane Spiegler, Alexander Humberg, Kathrin Hanke, Kirstin Faust, Julia Pagel, Leila Eyvazzadeh, Kim Brenner, Claudia Roll, Sabine Pirr, Dorothee Viemann, Dimitra Stavropoulou, Philipp Henneke, Birte Tröger, Thorsten Körner, Anja Stein, Christoph Derouet, Michael Zemlin, Christian Wieg, Jan Rupp, Egbert Herting, Wolfgang Göpel, Christoph Härtel

**Affiliations:** 1 Department of Pediatrics, University of Lübeck, 23562 Lübeck, Germany; janina.marissen@uksh.de (J.M.); bastian.siller@uksh.de (B.S.); uni@dr-spiegler.de (J.S.); alexander.humberg@uksh.de (A.H.); kathrin.hanke@uksh.de (K.H.); kirstin.faust@uksh.de (K.F.); julia.pagel@uksh.de (J.P.); leila.eyvazzadeh@gmail.com (L.E.); kim.brenner@uksh.de (K.B.); Egbert.Herting@uksh.de (E.H.); wolfgang.goepel@uksh.de (W.G.); christoph.haertel@uksh.de (C.H.); 2 German Center for Infection Research (DZIF), Partner Site Hamburg-Lübeck-Borstel-Riems, 38124 Braunschweig, Germany; jan.rupp@uksh.de; 3 Department of Pediatrics, Vestische Children’s Hospital Datteln, 45711 Datteln, Germany; c.roll@kinderklinik-datteln.de; 4 Department of Neonatology, Hannover Medical School, 30159 Hannover, Germany; pirr.sabine@mh-hannover.de (S.P.); viemann.dorothee@mh-hannover.de (D.V.); 5 Center for Pediatrics and Adolescent Medicine, Medical Center and Medical Faculty, University of Freiburg, 79098 Freiburg, Germany; dimitra.stavropoulou@uniklinik-freiburg.de (D.S.); philipp.henneke@uniklinikum-freiburg.de (P.H.); 6 Institute for Immunodeficiency, Medical Center and Medical Faculty, University of Freiburg, 79098 Freiburg, Germany; 7 Children’s Hospital Links der Weser Bremen, 28277 Bremen, Germany; birte.troeger@gesundheitnord.de (B.T.); thorsten.koerner@klinikum-bremen-ldw.de (T.K.); 8 Department of Neonatology and General Pediatrics, University of Essen, 45147 Essen, Germany; anja.stein@uk-essen.de; 9 Department of Neonatology and General Pediatrics, Saar University of Homburg, 66424 Homburg, Germany; christoph.derouet@uks.eu (C.D.); michael.zemlin@uks.eu (M.Z.); 10 Children’s Hospital Aschaffenburg-Alzenau, 63739 Aschaffenburg, Germany; christian.wieg@klinikum-ab-alz.de; 11 Department of Infectious Diseases and Medical Microbiology, University of Lübeck, 23562 Lübeck, Germany

**Keywords:** probiotic prophylaxis, human milk, prematurity, sepsis, growth failure

## Abstract

**Objective**: To evaluate the nutrition-related effects of prophylactic *Lactobacillus acidophilus/Bifidobacterium infantis* probiotics on the outcomes of preterm infants <29 weeks of gestation that receive human milk and/or formula nutrition. We hypothesize that human-milk-fed infants benefit from probiotics in terms of sepsis prevention and growth. Methods: We performed an observational study of the German Neonatal Network (GNN) over a period of six years, between 1 January, 2013 and 31 December, 2018. Prophylactic probiotic use of *L. acidophilus/B. infantis* was evaluated in preterm infants <29 weeks of gestation (*n* = 7516) in subgroups stratified to feeding type: (I) Exclusively human milk (HM) of own mother and/or donors (HM group, *n* = 1568), (II) HM of own mother and/or donor and formula (Mix group, *n* = 5221), and (III) exclusive exposure to formula (F group, *n* = 727). The effect of probiotics on general outcomes and growth was tested in univariate models and adjusted in linear/logistic regression models. Results: 5954 (76.5%) infants received *L. acidophilus/B. infantis* prophylactically for the prevention of necrotizing enterocolitis (NEC). Probiotic use was associated with improved growth measures in the HM group (e.g., weight gain velocity in g/day: effect size B = 0.224; 95% CI: 2.82–4.35; *p* < 0.001) but not in the F group (effect size B = −0.06; 95% CI: −3.05–0.28; *p* = 0.103). The HM group had the lowest incidence of clinical sepsis (34.0%) as compared to the Mix group (35.5%) and the F group (40.0%). Only in the Mix group, probiotic supplementation proved to be protective against clinical sepsis (OR 0.69; 95% CI: 0.59–0.79; *p* < 0.001). Conclusion: Our observational data indicate that the exposure to *L. acidophilus/B. infantis* probiotics may promote growth in exclusively HM-fed infants as compared to formula-fed infants. To exert a sepsis-preventive effect, probiotics seem to require human milk.

## 1. Introduction

Probiotics that act as gut colonizers of human-milk-fed infants [1] have a high potential to foster the early microbiome development [2]. Thus, they might prevent dysbiosis-associated complications such as necrotizing enterocolitis (NEC) and sepsis [3,4]. Numerous studies on the therapeutic effects of probiotics in preterm infants have been performed [4]. However, the results remain inconclusive due to a high variability in study protocols, target populations and endpoints, probiotic formulations (e.g., strain composition and inclusion of single vs. multiple strains), and the context of nutrition [5]. After the publication of several meta-analyses proposing a benefit for preterm infants’ short-term outcomes, prophylaxis with *Bifidobacterium longum* and *Bifidobacterium infantis/Lactobacillus acidophilus* has been adopted into clinical routine by many European neonatal intensive care units (NICUs), for example in Austria [6], the Netherlands [7], and Germany [8]. In hospitals of the German Neonatal Network (GNN), the implementation of probiotic use in 2009/2010 led to a decrease in NEC incidence in infants discharged in 2011 and 2012 after the change of strategy [8]. Despite the use of probiotics in >80% of extremely low-birth-weight infants (ELBWI), however, NEC and sepsis still remain significant causes of morbidity and mortality in this vulnerable population [9]. To promote a more personalized medical approach to preterm babies, there is an urgent need to define those populations who would benefit most from probiotic prophylaxis. Recent data suggest that the type of enteral feeding (breastmilk or bovine-based formula) modifies the effects of probiotics in preterm infants [6,7]. In line with this, the nutritional content of human milk oligosaccharides (HMOs)—a major metabolic source for bifidobacteria—was found to be predictive for the NEC risk [10]. Human milk contains numerous immune-related compounds such as leukocytes, lysozymes, nucleotides, and cytokines [11], whereas HMOs were found to directly mediate the prebiotic effect of bifidobacterial growth [12].

Herein we hypothesize that human milk as a nutritional source is required for probiotics to provide a sepsis-preventive and growth-promoting effect. Accordingly, we performed an observational study in a large GNN cohort of extremely preterm infants <29 weeks of gestation discharged after the year 2012 and evaluated the impact of probiotics in the context of feeding strategies.

## 2. Methods

### 2.1. The German Neonatal Network

The German Neonatal Network (GNN; www.vlbw.de) is a population-based observational multicenter cohort study enrolling Very low birth weight infants (VLBWI) at 64 neonatal intensive care units (NICUs) in Germany. Within the study period, data were collected from infants discharged between 1 January, 2013 and 31 December, 2018. Preterm infants of a birth weight <1500 g and/or a gestational age between 22 + 0 and 28 + 6 weeks, who were actively managed with intensive care, met the inclusion criteria. Infants with lethal malformations or those treated with comfort (palliative) care were excluded from the study. In the analysis of this study, only cases with complete documentation for feeding type were included.

After obtaining written informed parental consent, predefined data on general neonatal characteristics and antenatal and postnatal treatment and outcome were recorded for each patient on clinical record files at the participating centers. After discharge, data sheets were sent to the study center (University of Lübeck). Data quality was evaluated by a physician trained in neonatology via annual on-site monitoring of completed record files. After monitoring, data were coded and evaluated. 

### 2.2. Prophylactic Probiotic Supplementation

The probiotic formulation consisting of *B. infantis.* and *L. acidophilus* corresponds to the formulation that has been most commonly used among the participating study sites in the past [8]. Probiotics were provided once or twice daily in capsules beginning from day 1 to 3 of life until day 28 of life. The recommended daily dose contained 1–3 × 10^9^ CFU (Colony forming units) *L. acidophilus* and 1–1.5 × 10^9^ *B. infantis*.

### 2.3. Subgroups Stratified to Type of Milk Feeding

The preparation (pasteurization, freezing, storage) of human milk (own mother’s and donor’s milk) before use was carried out according to local standards (e.g., cytomegalovirus sero-prevalence of mother) at the study site. In all centers providing donor milk, the samples were pasteurized before use for feeding. Three “human milk feeding” strata were applied:

I HM (HM group): Infants who were exclusively fed with own mother‘s and/or donor‘s milk.

II Mix (Mix group): Infants who were fed with HM and formula at any time during the primary stay in hospital.

III Formula (F group): Infants who were exclusively fed with formula.

### 2.4. Definitions

Gestational age was calculated from the “best obstetric estimate”. This is defined as the estimate of the infant’s gestation based on the birth attendant’s final estimate by using early prenatal ultrasound and obstetric examination [13].

Small-for-gestational age (SGA) was defined as a birth weight less than the 10th percentile for gestational age according to gender-specific standards for birth weight by gestational age in Germany [14].

Full enteral feeding was defined as enteral nutrition at a minimum of 150 mL/kg body weight per day.

Weight gain velocity was defined as gain in body weight, calculated as g/day (difference of the parameter at birth and at discharge/number of days in hospital). Growth velocity of body length was defined as gain in body length, calculated as mm/day (difference of the parameter at birth and at discharge/number of days in hospital). Head growth velocity was defined as gain in head circumference, calculated as mm/day (difference of the parameter at birth and at discharge/number of days in hospital). *Z*-scores are numerical measurements of the value’s relationship to the mean of the group values measured in terms of standard deviations from the mean (between −3.0 and 3.0). *Z*-scores were calculated for birth weight according to the 2003 Fenton preterm growth chart [15,16].

Clinical sepsis was defined as condition with at least two signs of systemic inflammatory response (temperature >38 °C or <36.5 °C, tachycardia >200/min, new onset or increased frequency of bradycardias or apneas, hyperglycemia >140 mg/dL, base excess <−10 mval/L, changed skin color, increased oxygen requirements), one laboratory sign (e.g., C-reactive protein >20 mg/L, immature/total neutrophil ratio >0.2, white blood cell count <5/nL), and the neonatologist’s decision to treat with anti-infective drugs for at least 5 days but no proof of causative agent in blood culture [17]. Blood culture confirmed sepsis was defined as clinical sepsis with proof of causative agent in the blood culture. If coagulase-negative *Staphylococcus* (CoNS) was isolated as a single pathogen in one peripheral blood culture, two clinical signs and one laboratory sign were required for classification of CoNS sepsis [17].

Bronchopulmonary dysplasia (BPD) was diagnosed when needing supplemental oxygen or ventilatory support at 36 weeks of postmenstrual age. Necrotizing enterocolitis (NEC) was defined as necrotizing intestinal inflammation requiring surgery, and focal intestinal perforation (FIP) was FIP requiring surgical treatment classified by the attending surgeon. Retinopathy of prematurity (ROP) was defined as typical retinal changes (ophthalmoscopy) requiring interventions such as laser therapy, cryotherapy, or intraocular vascular endothelial growth factor inhibitors.

### 2.5. Statistical Analyses

Data analyses were performed using the SPSS 24.0 data analysis package (Munich, Germany). Hypotheses in the univariate analysis were evaluated with Fisher’s exact test and Mann–Whitney U test. Only two-sided tests were used. A *p*-value of <0.05 was considered as statistically significant for single tests. 

Subsequent to univariate analyses, we included parameters with a *p*-value < 0.1 in multivariate logistic regression models and known confounders as independent variables: gestational age per week, gender, multiples, and SGA status. Odd ratios and 95% confidence intervals were calculated in order to identify the influence of probiotic prophylaxis on outcomes independent of the abovementioned confounders. The following outcome parameters were tested in multivariate models: necrotizing enterocolitis, retinopathy of prematurity requiring intervention, bronchopulmonary dysplasia, clinical sepsis, and blood culture confirmed sepsis. To address the problem of multiple comparisons, we performed Bonferroni corrections for multivariate analyses in order to protect from statistical Type I errors. Additional information derived from Bonferroni correction is indicated in Table 1 and Table 2 accordingly. In addition, we tested nested models for our analyses by calculating the Akaike information criterion (AIC) for all multivariate calculations in order to estimate relative quality for each model.

In order to evaluate the influence of probiotic prophylaxis on growth parameters, we conducted linear regression models using known/probable confounders as independent variables: gestational age per week, birth weight, gender, multiple birth, and maternal descent. Effect size and 95% confidence intervals (CI) were calculated. The following outcome parameters were tested in linear models: *Z*-score-based body weight at discharge according the 2003 Fenton growth chart for preterm infants, z-score-based weight gain, growth velocity of body weight (g/day), body length (mm/day) and head circumference (mm/day). A *p*-value of <0.05 was considered statistically significant. For primary and subgroup analyses, we used a uniform dataset with available data for all metric parameters. Infants with incomplete data for variables that were used in our analyses were not included.

Graphical analysis was carried out using GraphPad Prism (Version 6.00, GraphPad Software, La Jolla, CA, USA).

### 2.6. Ethical Approval

All study parts were ethically approved by the University of Lübeck Ethical Committee and the committees of the participating centers (vote no. 08-022). Informed consent was obtained from all subjects. All methods were carried out in accordance with relevant guidelines and regulations, specifically: the Declaration of Helsinki, the current revision of ICH (The International Council for Harmonisation) Topic E6, the Guidelines for Good Clinical Practice, and the Guidelines of the Council for International Organization of Medical Sciences, the WHO (World Health Organization) (“Proposed International Guidelines for Biomedical Research Involving Human Subjects”).

## 3. Results

From 1 January, 2013 until 31 December, 2018, 7516 extremely low gestational age neonates (ELGANs) were discharged in 64 GNN centers. The study cohort had a mean gestational age at birth of 26.5 weeks (median 26.7 weeks; SD 1.6 weeks) and a mean birth weight of 855 g (median 845 g; SD 248 g, Table 3). Patients were hospitalized for a median of 85 days. Moreover, 1568 infants (20.9%) were exclusively fed with human milk (HM) during their primary stay, 5221 infants (69.5%) received both HM of mothers/donors and formula (Mix group), and 727 infants (9.6%) were fed exclusively formula nutrition (F group).

### 3.1. Human Milk Feeding Has Increased in GNN Centers from 2013 to 2018

**Figure 1:**
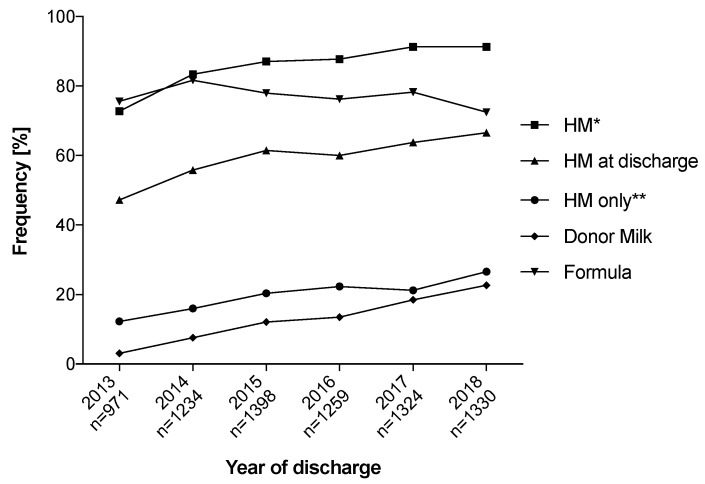
Changes in enteral feeding on German Neonatal Network (GNN) neonatal intensive care units (NICUs) between 2013 and 2018. HM, human milk; * includes all infants that were exposed to HM; ** includes all infants that were fed exclusively HM (own mother and/or donor).

To account for time trends in enteral feeding practices and current developments in human milk banks, we evaluated the proportion of infants receiving human milk (own-mother; donor milk), mix, or exclusively formula according to the year of the infant’s discharge. There has been an increasing rate of ELGANs that receive HM (72.8% in 2013 versus 91.3% in 2018; Figure 1) during primary stay in hospital and at discharge (47.2% in 2013 versus 66.6% in 2018). The administration of donor milk started in 2013 in 3.1% of infants and increased to 22.7% in 2018.

### 3.2. Formula-Fed Infants Have an Increased Risk for Adverse Short-Term Outcomes

In Table 3, the clinical characteristics of the groups are described with no major differences between the groups. *L. acidophilus/B. infantis* probiotics were administered prophylactically to 5682 ELGAN (76.5%) infants, with a frequency of 74.8% for infants receiving HM (HM group) and 74.9% for those with formula (F group; *p* = 0.935). Notably univariate analyses revealed that the F group had significantly higher rates of NEC (6.2% vs. 3.1%; *p* < 0.001), clinical sepsis (40.2% vs. 34.0%; *p* = 0.004), and severe ROP (6.5% vs. 3.2%; *p* = 0.004) as compared to the HM group. Exclusively formula-fed infants (F group) had a higher body weight at discharge and weight gain/day, head circumference, and body length than infants receiving HM in univariate analyses (*p* < 0.001, Table 4).

### 3.3. Probiotics Reduce the Risk for Clinical Sepsis in Infants with Human Milk and Formula Exposure

In the HM group, probiotics were not associated with reduced risk for adverse outcomes such as sepsis or BPD. In the Mix group, probiotics were associated with a risk reduction for clinical sepsis compared to untreated infants (univariate: 33.9% vs. 41.3%, *p* < 0.001; logistic regression: OR 0.69; 95% CI: 0.59–0.79, *p* < 0.001; Table 1 and Table 5). There were no significant effects of probiotics on the risk of NEC, ROP, sepsis, and BPD in the F group.

### 3.4. Probiotics Have a Growth-Promoting Effect in Exclusively Human-Milk-Fed Infants

A major effect of probiotic administration on growth was only observed in the HM group, i.e., higher body weight at discharge including Fenton *z*-scores (−1.49 vs. −2.13; *p* < 0.001), *z*-score-based weight gain (−1.30 vs. −1.83; *p* < 0.001), and growth rates of body weight, body length, and head circumference (Table 6). Weight gain velocity of HM infants (20.7 g/day) almost reached levels of mix (21.5 g/day), and formula-fed infants (21.9 g/day). In order to address confounding factors such as gestational age (catch-up growth of smaller infants), we performed a linear regression model including gestational age, birth weight, gender, multiple birth, and maternal descent (Table 2). In HM infants, probiotics were associated with higher bodyweight at discharge (effect size B = 0.261; 95% CI: 0.48–0.71; *p* < 0.001) and *z*-score-based weight gain. Notably probiotic treatment was associated with increased weight gain velocity (effect size B = 0.224; 95% CI: 2.82–4.35; *p* < 0.001), body length, and head circumference. This (probiotic) effect was observed to a lesser extent in the Mix group (body weight at discharge, head growth velocity), while Formula infants were not affected by probiotics in that aspect (Table 2).

## 4. Discussion

Our large-scale population-based data support the hypothesis that the source of enteral feeding has an impact on the effects of *L. acidophilus/B. infantis* probiotics in highly vulnerable preterm infants. In this context, human milk exposure is required for probiotics to provide a sepsis-preventive and growth-promoting effect. Exclusively formula-fed infants did not benefit from the administration of probiotics in terms of sepsis, NEC, or BPD risk.

The feeding of preterm infants with human milk has been previously associated with reduced morbidity and mortality [18,19,20,21]. The stabilization of the early host–microbiome interaction has been proposed as a crucial underlying mechanism for this beneficial effect [12]. On the other hand, human milk might not fully meet the nutritional requirements of preterm infants [22], which results in less weight gain in human-milk-fed preterm babies as compared to formula-fed infants [23]. Bovine multicomponent fortification of human milk has been proposed to cause inflammation [24], and there is a lack of scientific evidence about whether or not its routine use can impact growth and other short- and long-term outcomes [25]. Furthermore, there is uncertainty on whether pasteurized donated human milk should be preferred to preterm formula [26]. In clinical reality, most babies in participating GNN units receive a mix of human milk and formula while donor human milk availabilities are increasingly being established but still limited. We noted a temporal trend to higher rates of human milk feeding, which approached 90% in 2018. In such a setting with low NEC rates <4% in babies <29 weeks and 75% exposure to probiotics, we expected no further risk reduction for NEC by probiotics as compared to our previous findings before/after the introduction of probiotics into clinical routine [8]. In the current cohort study, we noted a promoting effect of probiotics on weight gain and growth velocities in HM-fed infants. Previous studies revealed inconsistent results that were not necessarily adjusted for the type of feeding [27,28,29,30]. A meta-analysis of 15 studies including 3751 infants <32 weeks and <1500 g birth weight demonstrated no significant effects of probiotics on weight gain [31]. In comparison to our cohort, the gestational age in this pooled study cohort analysis was higher while mean weight gain/day was lower (16 g/day vs. 21 g/day) indicating that the effects of probiotics are context sensitive. While the huge variability in study designs has been acknowledged, the authors conclude that probiotics are more effective in reducing morbidity when taken in human milk or human milk plus formula form, consumed for <6 weeks, administered at a dosage of <10^9^ CFU/d, and include multiple strains. We assume that promoted weight gain in HM-fed infants is correlated with the dynamics of the establishing intestinal microbiota, nutrient utilization, and immune–metabolome interaction [4,32]. *L. acidophilus/B. infantis* may require the complex composition of human milk to exert a growth-promoting effect and to stabilize gut immunity in order to prevent translocation sepsis [6,7,33]. Human milk includes endogenous probiotics, prebiotic carbohydrates, stem cells, and a concert of bioactive human milk factors (e.g., S100 A8/9 [34]) that have direct or indirect effects on the vulnerable host–gut microbiota interplay [11]. Hence, the sepsis-preventive effect of human milk may not be additionally increased by probiotics in exclusively HM-fed infants. Infants who are exposed to human milk and formula benefit from probiotics, which would compensate for the reduced abundance of bifidobacteria and lactobacilli in their gut microbiota composition [20,35]. Both, human milk feeding and probiotics might also be able to “reverse” antibiotic-induced gut dysbiosis [36], which needs to be subject to further long-term studies of extremely preterm infants.

### Strengths and Limitations

The major strengths of our study design are the large sample size and high quality of the clinical data that are monitored on-site by a study team trained in neonatology. The main limitations are the observational design, the lack of information on the daily type of feeding in the Mix group, indication for supplementation, and timing with bovine and individual fortification of human milk or formula. Furthermore, we did not have exact data on the timing of probiotics, the number of pasteurized milk portions, and the bacterial load of human own-mother milk (if not pasteurized) in the individual infants. Whether probiotics or the probiotic/prebiotic load of human milk is causal for the observed effects, needs to be addressed in future studies including extensive sequencing of human milk microbiome and the gut microbiome of the milk (+probiotic)-fed infants.

## 5. Conclusions

Evidence of nutrition-related effects of probiotic prophylaxis in preterm infants is scarce. In a large cohort of VLBWI, we conclude that supplementation of *L. acidophilus/B. infantis* and feeding strategies interact and have the potential of improving outcomes and growth in preterm infants. Our data demonstrates sepsis-preventive and growth-promoting effects exclusively in infants receiving human milk, supporting usage of human milk (including human milk from donors) in preterm infants whenever possible. Randomized, placebo-controlled trials as the PRIMAL clinical study [37] are pending to test hypothesis generating observational studies and to evaluate long-term effects of probiotics.

## Figures and Tables

**Table 1:** Effect of probiotic treatment on short-term outcomes in the context of feeding types.

	I HM	II Mix	III Formula
Surgery for NEC	OR 1.37 (95% CI: 0.69–2.73) *p* = 0.38	OR 0.84 (95% CI: 0.59–1.15) *p* = 0.26	OR 0.89 (95% CI: 0.5–2.0) *p* = 0.9
Clinical sepsis	OR 0.95 (95% CI: 0.73–1.22) *p* = 0.67	OR 0.69 (95% CI: 0.59–0.79) *p* < 0.001 *	OR 1.20 (95% CI: 0.9–1.7) *p* = 0.243
Sepsis (BC positive)	OR 1.09 (95% CI: 0.78–1.53) *p* = 0.60	OR 0.89 (95% CI: 0.74–1.06) *p* = 0.19	1.10 (95% CI: 0.7–1.8) *p* = 0.662
ROP	OR 1.51 (95% CI: 0.73–3.10) *p* = 0.27	OR 1.04 (95% CI: 0.72–1.51) *p* = 0.83	OR 1.35 (95% CI: 0.63–2.94) *p* = 0.44
BPD	OR 0.86 (95% CI: 0.65–1.14) *p* = 0.31	OR 0.90 (95% CI: 0.77–1.05) *p* = 0.19	1.31 (95% CI: 0.87–1.96) *p*= 0.19

HM, human milk, NEC, necrotizing enterocolitis, ROP, retinopathy of prematurity, BPD, bronchopulmonary dysplasia; NEC, necrotizing enterocolitis. Logistic regression analyses were performed by using the following independent variables: gestational age, multiple birth, gender, SGA, and treatment with probiotics. * Bonferroni correction did not change significance of the *p*-value.

**Table 2:** Effect of probiotics on growth parameters of the GNN cohort during their primary stay in hospital.

	I HM	II Mix	III Formula
Body weight at discharge (z-score, Fenton)	B = 0.261 95% CI: 0.48–0.71 *p* <0.001 *	B = 0.026 95% CI: 0.01–0.1 *p* = 0.029 ^#^	B = 0.015 95% CI: −0.12–0.18 *p* = 0.72
Weight gain (z-score, Fenton)	B = 0.23 95% CI: 0.42–0.62 *p* < 0.001 *	B= 0.022 95% CI: −0.01–0.1 *p* = 0.078	B= 0.06 95% CI: −0.04–0.26 *p* = 0.14
Growth velocity (g/day)	B = 0.224 95% CI: 2.82–4.35 *p* < 0.001 *	B = 0.00 95% CI: −0.61–0.62 *p* = 0.98	B = −0.06 95% CI: −2.90–−0.45 *p* = 0.15
Growth velocity of body length (mm/day)	B = 0.179 95% CI: 0.13–0.24 *p* < 0.001 *	B = 0.019 95% CI: −0.01–0.04 *p* = 0.184	B = −0.012 95% CI: −0.11–0.08 *p* = 0.761
Head growth velocity (mm/day)	B = 0.117 95% CI: 0.05–0.12 *p* < 0.001*	B = 0.03 95% CI: 0.003–0.04 *p* = 0.023 ^#^	B = -0.002 95% CI: −0.08–0.07 *p* = 0.966

HM, human milk. Growth velocity, weight gain, and growth of body length and head circumference were calculated by differences between parameters at birth and respective measures at discharge/number of days (duration of stay). Linear regression analyses were performed by using the following independent variables: gestational age, birth weight, multiple birth, gender, maternal descent, and exposure to probiotic prophylaxis within the three subgroups. * Bonferroni correction did not change significance of the p-value. ^#^ Not significant after Bonferroni correction.

**Table 3:** Clinical characteristics of the GNN cohort stratified to type of milk feeding.

	I HM	II Mix	III Formula	*p*-Value (HM vs. Formula)	Total
Number of infants *n*, (%)	1568, (20.9)	5221, (69.5)	727, (9.6)		7516, (100)
Gestational age (weeks)	26.4/1.68 (26.57)	26.52/1.61 (26.7)	26.6/1.95 (26.9)	0.024 ^#^	26.5/1.63 (26.7)
Birth weight (g)	841/257 (830)	861/245 (850)	858/243 (850)	0.067 ^#^	855/248 (845)
*Z*–score (birth weight)	−0.25/0.98 (−0.15)	−0.20/−0.92 (0.13)	−0.25/−0.16 (0.03)	0.661 ^#^	−0.22/0.93 (−0.14)
Gender, male (%)	54.4	52.9	52.4	0.380	53.2
Multiples (%)	30.9	34.4	26.1	0.020	33.1
SGA (%)	14.6	12.1	13.6	0.526	12.9
Caesarean section (%)	88.4	90.8	87.3	0.471	89.9
Vaginal delivery (%)	11.6	9.2	12.7	0.471	10.1
Hospitalization (days)	83/39 (79)	87/38 (81)	85/45 (80)	0.372 ^#^	85/40 (80)
Time to full enteral feeds (days)	18.5/14.6 (14.0)	17.7/14.3 (14.0)	19.8/1 (17.8)	0.341 ^#^	18.2/15.1 (14.0)
Duration of intravenous line (days)	26.0/23.7 (18.0)	26.2/25.1(18.0)	28.1/27.6 (18.0)	0.779 ^#^	26.4/25.3 (18.0)

HM, human milk; SGA, small-for-gestational-age (<10th Voigt percentile); *p*-values were derived from chi-square test, if not otherwise indicated (^#^, Mann–Whitney-U test). Continuous variables and *z*-scores are shown as mean/SD (median).

**Table 4:** Treatment, outcomes, and growth parameters of the GNN cohort stratified to type of milk feeding.

	I HM	II Mix	III Formula	*p*-Value (HM vs. Formula)	Total
Number of infants *n*, (%)	1568 (20.9)	5221 (69.5)	727 (9.6)		7516 (100)
Antibiotic treatment (%)	94.3	93.5	95.3	0.323	93.7
Probiotic prophylaxis (%)	74.8	78.3	74.9	0.935	76.5
Surgery for NEC (%)	3.1	3.8	6.2	<0.001 ^#^	3.9
BC-confirmed sepsis (%)	14.6	16.8	15.7	0.506	16.3
Clinical sepsis (%)	34.0	35.5	40.2	0.004	35.6
BPD (%)	26.4	28.5	28.7	0.240	28.0
Severe ROP (%)	3.2	3.6	6.5	0.004	3.9
Weight (g) at discharge	2460/750 (2460)	2711/733 (2650)	2752/1020 (2731)	<0.001 ^#^	2653/818 (2615)
*Z*-score (birth weight)	−1.73/−1.63 (0.03)	−1.35/−1.32 (0.85)	−1.22/−1.22 (0.79)	<0.001 ^#^	−1.40/0.88 (−1.36)
Weight gain velocity (g/day)	19.1/ 6.3 (19.7)	21.5/ 9.3 (21.6)	22.2/ 10.1 (22.4)	<0.001 ^#^	21.1/9.09 (21.4)
Weight at discharge (z-scores, Fenton)	−1.53/1.42 (0.03)	−1.16/−1.12 (0.83)	−1.03/−0.99 (0.79)	<0.001 ^#^	−1.2/0.86 (−1.15)
Growth velocity of body length (mm/day)	1.36/0.44 (1.37)	1.43/0.39 (1.42)	1.49/0.48 (1.45)	<0.001 ^#^	1.42/0.41 (1.41)
Head growth velocity (mm/day)	0.98/0.37 (0.98)	1.03/0.28 (1.03)	1.06/0.40 (1.02)	<0.001 ^#^	1.02/0.31 (1.02)

HM, human milk; BPD, bronchopulmonary dysplasia; NEC, necrotizing enterocolitis; ROP, retinopathy of prematurity; BC, blood culture. Growth velocity and weight gain were calculated by differences between parameters at birth and respective measures at discharge/number of days (duration of stay). Continuous variables and z-scores are shown as mean/SD (median); *p*-values were derived from Fisher’s exact test or Mann–Whitney-U test if indicated with ^#^.

**Table 5:** Outcomes of the GNN cohort stratified to type of milk feeding and treatment with probiotics.

	I HM		*p*	II Mix		*p*	III Formula		*p*
	No Probiotics	Probiotics		No Probiotics	Probiotics		No Probiotics	Probiotics	
Number of infants *n*, (%)	395 (25.2)	1173 (74.8)		1135 (21.7)	4086 (78.3)		182 (25.1)	545 (74.9)	
NEC (%)	2.8	3.3	0.65	4.4	3.6	0.21	7.2	5.9	0.54
Clinical sepsis (%)	36.6	33.2	0.02	41.3	33.9	<0.001^#^	37.0	41.0	0.35
BC-confirmed sepsis (%)	14.6	14.6	0.98	18.2	16.5	0.17	15.0	15.8	0.79
Severe ROP (%)	2.7	3.5	0.45	3.8	3.6	0.76	2.9	7.4	0.34
BPD	30.5	25.0	0.003	29.5	28.2	0.38	26.5	29.3	0.48

HM, human milk; BPD, bronchopulmonary dysplasia; NEC, necrotizing enterocolitis; ROP, retinopathy of prematurity; BC, blood culture. Continuous variables and *z*-scores are shown as mean/SD (median). Categorical variables are shown as percent. *p*-values were derived from Fisher’s exact test or Mann–Whitney-U test if indicated with ^#^.

**Table 6:** Growth parameters of the GNN cohort stratified to type of milk feeding and treatment with probiotics.

	I HM		*p*	II Mix		*p*	III Formula		*p*
	No Probiotics	Probiotics		No Probiotics	Probiotics		No Probiotics	Probiotics	
Weight (g) at discharge ^#^	2213/782 (2190)	2542/721 (2535)	<0.001	2692/833 (2670)	2716/756 (2640)	0.99	2455/1128 (2540)	2844/958 (2800)	<0.001
Weight at discharge; *z*-score, Fenton ^#^	−2.13/1.13 (−2.11)	−1.49/0.87 (−1.43)	<0.001	−1.36/0.91 (−1.31)	−1.35/0.83 (−1,33)	0.79	−1.29/0.83 (−1.19)	−1.21/.78 (−1.23)	0.64
Weight gain (*z*-scores, Fenton)	−1.83/1.16 (−1.72)	−1.30/0.83 (−1.27)	<0.001	−1.23/0.91 (−1.13)	−1.14/0.80 (−1.12)	0.05	−1.12/0.82 (−1.06)	−1.01/0.78 (−0.99)	0.51
Growth velocity (g/day) ^#^	18.81/7.37 (16.19)	20.69/6.63 (20.87)	<0.001	21.48/13.10 (21.60)	21.49/7.97 (21.62)	0.32	22.80/16.01 (22.00)	21.91/7.12 (22.47)	0.36
Head growth velocity (mm/day)	0.930/0.502 (0.89)	0.996/0.320 (1.0)	<0.001	1.012/0.332 (1.008)	1.11/0.261 (1.028)	0.017	1.058/0.389 (1.036)	1.056/0.400 (1.017)	0.89
Growth velocity (body length; mm/day)	1.210/0.442 (1.216)	1.403/0.429 (1.4)	<0.001	1.413/0.407 (1.406)	1.432/0.380 (1.422)	0.139	1.511/0.654 (1.451)	1.49/0.43 (1.452)	0.775

HM, human milk. Growth velocity, weight gain, and growth of body length and head circumference were calculated by differences between parameters at birth and respective measures at discharge/number of days (duration of stay). Continuous variables and z-scores are shown as mean/SD (median), *p*-values were derived from Mann–Whitney-U test if indicated with ^#^.

## Abbreviations

BPD	bronchopulmonary dysplasia
HM	human milk
ELGAN	extremely low gestational age neonates
FIP	focal intestinal perforation
GNN	German Neonatal Network
NEC	necrotizing enterocolitis
NICU	neonatal intensive care unit
ROP	retinopathy of prematurity
SGA	small for gestational age
